# Investigations on the Fusants From Wide Cross Between White-Rot Fungi and *Saccharomyces cerevisiae* Reveal Unknown Lignin Degradation Mechanism

**DOI:** 10.3389/fmicb.2022.935462

**Published:** 2022-07-11

**Authors:** Qi Shao, Xin Li, Ying Chen, Zhijun Zhang, Yong Cui, Huan Fan, Dongsheng Wei

**Affiliations:** ^1^Department of Microbiology, College of Life Sciences, Nankai University, Tianjin, China; ^2^Institute of Agro-Products Preservation and Processing Technology, Tianjin Academy of Agricultural Sciences, Tianjin, China; ^3^Tianjin Tianren Century Technology Co., Ltd., Tianjin, China; ^4^Institute of Animal Husbandry and Veterinary Research, Tianjin Academy of Agricultural Sciences, Tianjin, China

**Keywords:** lignin degradation, white-rot fungi, protoplast fusion, biomass conversion, 2D-HSQC NMR

## Abstract

The degradation of lignocellulose by fungi, especially white-rot fungi, contributes a lot to carbon cycle, bio-fuel production, and many other bio-based applications. However, the existing enzymatic and non-enzymatic degradation mechanisms cannot be unequivocally supported by *in vitro* simulation experiment, meaning that additional mechanisms might exist. Right now, it is still very difficult to discover new mechanisms with traditional forward genetic approaches. To disclose novel lignin degradation mechanisms in white-rot fungi, a series of fusants from wide cross by protoplast fusion between *Pleurotus ostreatus*, a well-known lignin-degrading fungus, and *Saccharomyces cerevisiae*, a well-known model organism unable to degrade lignocellulose, was investigated regarding their abilities to degrade lignin. By analyzing the activity of traditional lignin-degrading enzyme, the ability to utilize pure lignin compounds and degrade corn stalk, a fusant D1-P was screened out and proved not to contain well-recognized lignin-degrading enzyme genes by whole-genome sequencing. Further investigation with two-dimension nuclear magnetic resonance (NMR) shows that D1-P was found to be able to degrade the main lignin structure β-O-4 linkage, leading to reduced level of this structure like that of the wild-type strain *P. ostreatus* after a 30-day semi-solid fermentation. It was also found that D1-P shows a degradation preference to β-O-4 linkage in A_β_(S)-*threo*. Therefore, wide cross between white-rot fungi and *S. cerevisiae* provides a powerful tool to uncover novel lignocellulose degradation mechanism that will contribute to green utilization of lignocellulose to produce bio-fuel and related bio-based refinery.

## Introduction

Carbon dioxide is collected from atmosphere and stored into plant, the huge carbon sink on the earth. How does carbon dioxide cycle back to the atmosphere really matters for carbon neutralization (Brienen et al., 2020). Lignocellulose, in the form of plant cell wall, is the major carbonic substance in this sink. Therefore, converting lignocellulose into carbon dioxide is the main pathway to maintain carbon neutralization. The major natural players in this converting process are a group of fungi, wood-degrading fungi that belong mostly to Basidiomycetes and Ascomycetes (Janusz et al., 2017).

Wood-degrading fungi have long been proposed to utilize enzymatic and non-enzymatic reactions to degrade recalcitrant lignin protector, and the exposed cellulose would be consumed by hydrolases in various microbes afterward (ten Have and Teunissen, 2001; Janusz et al., 2017). Therefore, breaking lignin barrier in lignocellulose was the first and the most important step toward carbon cycling. The mechanism involved in the lignin barrier breaking is not only essential for carbon recycling but also for many bio-based refineries. Fenton reaction was proposed to be responsible for non-enzymatic cleavage or degradation of lignin component (Goodell, 2003). Lignin-degrading enzymes such as laccase, manganese peroxidase, versatile peroxidase, and lignin peroxidase were proposed to enzymatically degrade lignin components (Hammel and Cullen, 2008; Alfaro et al., 2014; Pollegioni et al., 2015). Despite many progresses on elucidating mechanism of lignin degradation, there were still many mysteries or contradictions in this regard. For example, the involvement of well-recognized small mediator, veratryl alcohol, in the cleavage of lignin components has been questioned by Hammel and Cullen (2008). Regarding the ability of these wood-degrading fungi to convert lignocellulose into carbon dioxide, a recent investigation shows that these fungi can transform lignin into carbon dioxide (Del Cerro et al., 2021). However, whether this conversion depends on those enzymes mentioned above remains unclear (Houtman et al., 2018).

In addition, after so many years of investigation, there were very few investigations using proposed non-enzymatic and enzymatic mechanism to successfully and efficiently degrade the whole plant cell wall *in vitro* (Wariishi et al., 1991). Most investigations use model lignin compounds as the substrate whose structures were far more like the native lignin (Picart et al., 2017; Gall et al., 2018). These results indicate that some unknown factors or mechanism might still be there to be discovered. This proposition could be supported by the fact that, due to the existence of multiple isoform of laccase (Lac), manganese peroxidase (MnP), versatile peroxidase, and lignin peroxidase (LiP) genes in one-specific wood-degrading fungus might play redundant or distinct roles in the degradation of lignin (Janusz et al., 2017). The roles of these enzymes in lignin degradation need confirmation by gene knockout technology which, unfortunately, is still lacking due to the lack of multiple selection markers. Nakazawa et al. (2016) designed a 5-fluoroorotic acid and 5-fluorocytosine counter-selection method to recycle selection marker to generate multi-gene disruptions in *P. ostreatus*. However, there were very few investigations that have used this genetic method to prove the function of these enzymes (Mei et al., 2019). It is possible due to the tedious selection process in this method or other unknown factors.

Wide cross has been used in plant and animal breeding practice, even though some results were confusing (Cocking, 1984). According to our acknowledgment, it has never been used in fungi breeding. However, this method provides an interesting method to dissect or reveal novel lignin degradation mechanism under current situation. Through random combination of chromosomes from wood-degrading fungi and non-wood-degrading fungi, it would be possible to eliminate all known lignin-degrading related enzymes in a fusant. By carefully investigating the ability of these fusants to degrade lignin and next-generation sequencing technology, it is possible to screen out those lignin-degrading fusants without traditional enzymes. Following this reasoning, 29 fusants were obtained by wide cross between white-rot fungus *P. ostreatus* and *S. cerevisiae* by protoplasts electrofusion in previous works, and all these fusants exhibited filamentous form (Tu et al., 2013; Yan et al., 2015a,b,c).

In this work, the fusants were screened based on their ability to secrete traditional lignin degradation enzymes and the corresponding ability to degrade lignin and corn stalk. By the combination of enzyme analysis, next-generation sequencing, and chemical analysis of the degradation products of the corn stalk, one of the fusant D1-P was proved to be able to degrade the main lignin structure β-O-4 linkage with optical preference without the help of traditional lignin degradation enzymes, thus implying the involvement of new enzyme or mechanism in lignin degradation.

## Materials and Methods

### Materials and Chemicals

Corn stalk was provided by Tianjin Institute of Animal Husbandry and Veterinary Medicine, Tianjin, China. Cellulase (C6339, 50 U/mg), β-glucosidase from *Aspergillus niger* (G834635, 100 U/g), and xylanase (X875254, 6,000 U/mg) were purchased from Macklin (Shanghai China). Other chemicals used in this study were purchased from Sigma-Aldrich (Shanghai China) or Tianjin Chemical Reagent Factory (Tianjin China) unless otherwise specified. All chemicals were used without any further purification.

### Microorganisms

All fungal strains used in this study were obtained from Tianjin Institute of Animal Husbandry and Veterinary Medicine (Tianjin, China) including *P. ostreatus* 595 strain (WT) and 29 fusants (D1-P, D1-S, D1-Z, D2-2, D2-3, D2-4, D5, H2-1, H2-2, H2-3, H2-5, H2-7, H2-8, H2-9, H2-10, H2-11, 8-11, H1-A, H1-B, H1-C, H1-D, H1-G, H1-I, H1-L, H1-M, H1-N, H1-O, H1-X, and H1-Y) generated by protoplast fusion between *P. ostreatus* 595 and *S. cerevisiae*.

### Culture Medium

Potato dextrose agar (PDA) contained peeled potato (200 g/L), glucose (20 g/L), and agar (20 g/L). Calcium lignosulfonate solid medium (CLSS) contained calcium lignosulfonate (10 g/L) and agar (20 g/L). Alkaline lignin solid medium (ALS) contained alkaline lignin (10 g/L) and agar (20 g/L). Calcium lignosulfonate liquid medium (CLSL) contained only calcium lignosulfonate (6.25 g/L) in distilled water. Alkaline lignin liquid medium (ALL, pH 6.2) contained only alkaline lignin (0.1 g/L) in distilled water. Corn stalk medium was composed of 20 g Wiley-milled corn stalk and 60-mL distilled water. < containing 0.04 % (w/v) Remazol brilliant blue R (RBBR), 0.01% (w/v) guaiacol, and 0.50 % (w/v) tannic acid, respectively, was used to detect laccase activity.

### Growth Measurement of the Fusants on Different Solid Plates

Growth on PDA: A circular disk with diameter of 10 mm was cut from the edge of the actively growing colony on PDA plate. Then, the mycelial disk was inoculated at the center of a new PDA plate and incubated for 6 days at 28°C. The colony radius was then measured and compared with that of WT strain.

Growth on CLSS and ALS: Similar method was used to evaluate the growth of these fusants on these plates, except that the colony radius was measured after incubation for 7 days (CLSS) and 11 days (ALS) at 28°C, respectively.

Growth on corn stalk: The mycelium disk with same size was inoculated at the center of a cellophane on top of the PDA plate. After the whole cellophane was fully covered by mycelium, meaning approximately the same amount of mycelium was obtained, the mycelium was collected from the cellophane and broken into pieces in sterile water (100 ml/pcs) with a warring blender. The mycelium suspensions (25 mL) were mixed manually with Wiley-milled corn stalk (20 g) that has been sterilized three times in a 250-mL conical flask with 60-mL distilled water. The stalk was incubated at 28°C for 40 days or indicated time.

### Measurement of Ligninolytic Enzyme Activities

Three fresh mycelial disks with diameter of 5 mm were inoculated into 200 mL CLSL and ALL, respectively. Cultures were statically incubated at 28°C until visible mycelium mat was obtained. Crude enzyme in the supernatant was obtained by removing mycelium with centrifugation. MnP activity was determined using 2,6-dimethylphenol (2,6-DMP) as the substrate. The reactions occurred in the presence of 5 μL of 2,6-DMP, 50 μL of 100 mmol/L MgSO_4_, 50 μL enzyme extract, and 2 Ml of sodium tartrate buffer (pH 4.5), and 20 μL of 100 mmol/L H_2_O_2_ was added at last to start the reaction. Lac activity was determined using 2,2’-azino-bis (3-ethylbenzothiazoline-6-sulfonic acid) (ABTS) as the substrate. The reactions occurred in the presence of 500 μL 7 mmol/L ABTS, 500 μL enzyme extract, and 3 mL of sodium acetate buffer (pH 5.0).

Enzyme activity was calculated by the variation of absorbance at 469 nm (MnP) / 420 nm (Lac) using Equation 1 (Baltierra-Trejo et al., 2015).


(1) $$\frac{U}{L}=\frac{\Delta \,A\times {\text{V}}_{t}\times {\text{D}}_{f}\times 10^{6}}{t\times \varepsilon \times \text{d}\times {\text{V}}_{s}},$$

*where U/L* represented the enzymatic activity meaning the amount of enzyme converts 1 μmol substrate per minute per liter in the current reaction conditions. Δ*A* is the variation of absorbance. *V*_*t*_ means the total volume of reaction (ml). *D*_*f*_ is the dilution factor. *t* represented the time of reaction. ε is the molar extinction coefficient of oxidized 2,6-DMP/ABTS at 469 nm/420 nm (55,000 M**^–^**^1^⋅cm**^–^**^1^/36,000 M**^–^**^1^⋅cm**^–^**^1^). *d* is the optical path (1 cm). *V*_*s*_ is the sample volume (ml).

### Mass Spectrometry of the Secreted Protein

Four mycelium disks with diameter of 5 mm coming from fresh WT and fusant D1-P plates, respectively, were inoculated into 100 mL PD liquid medium and incubated statically for 7 days at 28°C. Then, the mycelium was collected from the culture by centrifugation at 5,000 rpm for 10 min and disrupted for 30 s with a high-speed warring blender in aseptic distilled water. The uniformly dispersed mycelium suspension (30 mL) was inoculated into a triangular bottle containing 5 g of corn stalk and cultivated at 28°C for 7 days, 14 days, and 21 days, respectively. Phosphate buffer (PBS, 0.1 mol/L, Ph 7.0) was supplemented into the culture, and the culture medium was gently washed for 30 min at each interval. Secreted protein was obtained by filtration through Miracloth (CALBIOCHEM, Darmstadt, Germany). Protein solution collected at different time point was pooled together and concentrated using a freeze dryer until it turned into powder to get WT mixed sample and D1-P mixed sample. The powder was dissolved with PBS buffer and used for SDS-PAGE electrophoresis to concentrate and eliminate non-protein impurities. After concentration, the single protein band containing all secreted protein was cut out from the gel. LC-MS/MS of the total secreted protein was done by Beijing Protein Innovation Co., Ltd., (Beijing, China) according to standard protocol. The original LC-MS/MS data were assigned with the database of *P. ostreatus* PC9 and PC15 (uni_pleurotus pleurotus).

### Genome Extraction and Preliminary Screening

WT and D1-P were cultured in PD medium at 28°C for 3 days. The mycelia were harvested by centrifugation at 8,000 rpm at 4°C for 10 min. Thereafter, the harvested mycelia were broken by bead-beating technique with lysis buffer. DNA was extracted using extract solution (Solarbio, Beijing, China) and purified with chloroform, isopropanol, and phenol–chloroform (Lee et al., 2021). Preliminary screening the lignin degradation enzyme genes was done by PCR. A total of 20 pair of primers representing all known lignin degradation enzyme in *P. ostreatus* PC9 and PC15 were designed with SnapGene software and used in PCR amplification (Supplementary Table 1). The amplified product was detected with electrophoresis on 0.8% agarose gel and recorded with a digital camera.

### Genome Sequencing of WT and Fusant D1-P

Genome sequencing and assembly were done by BGI (Shenzhen, China). As 595 strain was a dikaryotic strain, all PCR or DNA polymerase-based sequencing methods would lead to the production of chimeric artifacts. Therefore, DNA polymerase-independent Nanopore long reads were used for sequencing *P. ostreatus* 595 strain using PromethION platform. The long reads were *de novo* assembled with overlap–layout–consensus (OLC) algorithm with all-against-all pairwise alignment using Cano software. After that, short Illumina sequencing was used to correct the errors produced in nanopore sequencing (Lee et al., 2021). The error correction process is validated using Benchmarking Universal Single-Copy Orthologs (BUSCO) completeness scores.

Reads were assembled using variety of software, and it can be roughly divided into four parts: (1) Subreads correct; (2) Corrected Reads Assembly; (3) Correct single base; (4) Link Contig to Scaffold and fill gaps. Subreads correct was done using software (Pbdagcon and Falcon Consensus) to correct Subreads itself, or mix to correct Subreads with Proovread, and the corrected Subreads are more accurate and reliable. The Corrected Reads were assembled based on Corrected Reads using several software (Celera, Falcon), respectively, and then choose the best assembly result. Single base error was corrected in assembly result with NGS data using software GATK. Contigs were linked to scaffold with long inset size pair-end reads using SSPACE_Basic_v2.0, and the gap was then filled using software pbjelly2. Genomic component analysis was completed by homology (Genewise v2.20), SNAP (SNAP 2010-07-28), and Augustus (Augustus v3.2.1) using *P. ostreatus* PC9 and *P. ostreatus* PC15 as a reference genome including the following: (a) genome component; (b) analysis on repeat sequences; (c) non-coding RNA. The predicted ORFs are annotated by GO, KEGG, Swiss-Prot, CAZy, NR, and COG, respectively. The sequence information was submitted to NCBI with an accession number of SAMN23459969.

The second-generation draft genome sequencing of D1-P was completed by BIOZERON Co., Ltd., (Shanghai, China). Briefly, the genome was sonicated into small fragments (300–400 bp) to construct a genomic library. After PCR, the library was sequenced by Hiseq platform to perform a 2 × 150 bp seq. The data were evaluated by Trimmomatic v0.39, first assembled with ABySS v2.0.2 and second assembled with CANU v2.0. The genes were predicted by homology alignment combined with Denovo using Genewise v2.20. The predicted ORFs are annotated by SWISSPROT, COG, GO, KEGG, and NR, respectively. The sequence information was submitted to NCBI with an accession number of SAMN23459970. The procedure for the analyze of WT and D1-P is briefly illustrated in Supplementary Figure 1.

### Isolation of Lignin From Corn Stalk

Milled corn stalk lignin (MCSL): Dried stalks were ground using a Wiley mill under cooling conditions. Stalk particles were extracted with ethanol/benzene solution (1:2, v/v) for 48 h to obtain extractive-free samples, and the extracted stalk was air-dried and subsequently vacuum-dried. The vacuum-dried stalk was ball-milled in a planetary ball miller (MQ-SC-A, Qinhuangdao Taiji Ring Nano-Products, China) at 20°C for 3 h. After adjusting water content to 70%, the ball-milled sample was treated with WT and D1-P at 28°C for 15 weeks. Then, the ball-milled sample (control) and the treated samples were purified by precipitation with 90% (w/w) acetic acid. The purified ball-milled only sample was named as MCSL and used as a control sample for further test, and the other two treated samples were named as WMCSL (WT treated MCSL) and DMCSL (D1-P treated MCSL), respectively. The procedure for the isolation of MSCL from corn stalk is illustrated in Supplementary Figure 2.

Cellulolytic enzyme lignin (CEL): The Wiley-milled stalk was first treated by WT and fusant D1-P at 28°C for 4 weeks, and the treated samples were extracted with ethanol/benzene (1:2, v/v) for 48 h to obtain extractive-free samples. The ball milling treatment was performed as mentioned before. The ball-milled sample (5 g) was suspended in an enzyme cocktail containing cellulase, xylanase, and β-glucosidase with a ratio of 1 U: 1.2 U: 1 U in 100 mL of acetate buffer (pH 4.8) in a 500-ml conical flask. The enzymatic hydrolysis reaction was performed by incubation in a shaker at 50°C with 180 rpm for 72 h. After enzymatic hydrolysis, the supernatant was removed by centrifugation and the residues were washed by acetate buffer (pH 4.8) and deionized water for three times, respectively. The washed enzymatic residues were freeze-dried and purified with 90% (w/w) acetic acid. The purified enzymatic-hydrolysis-only sample was named as CEL as a control sample for further test, and the other two samples were named as WCEL and DCEL, respectively. The procedure for the isolation of CEL from corn stalk is illustrated in Supplementary Figure 2.

### High-Performance Liquid Chromatography

The content of cellulose, hemicellulose, and lignin in corn stalk was determined with high-performance liquid chromatography (HPLC) according to the National Renewable Energy Laboratory (NREL) standard method (Sluiter et al., 2008). Cellulose and hemicellulose were determined by HPLC with a chromatographic column Aminex HPX-87H. The analysis conditions were as follows: H_2_SO_4_ (5 mmol/L), 0.6 ml/min flow rate, 65°C column temperature, 50°C differential refractive index temperature. The percentage of lignocellulose and degradation rate was calculated with the following formula (2), (3):


(2) $$Percentageoflignocelluloe\left(\%\right)=\text{[}M_{n}/M_{t}]\times 100\%,$$

(3) $$D\,e\,g\,r\,a\,d\,a\,t\,i\,o\,n\,r\,a\,t\,e=\left[\left(M_{0}-M_{n}\right)/M_{0}\right]\times 100\%,$$

*where M_*t*_* is the total weight of degraded residue, *M*_0_ is the initial lignocellulose weight, and *M*_*n*_ is the sampling lignocellulose residue weight after degradation.

### Fourier Transform Infrared Spectroscopy

Fourier transform infrared spectroscopy (FTIR) spectra of MCSL, WCSL, and DCSL were acquired on a Thermo Fisher Scientific Nicolet iS50 spectrophotometer. Samples were analyzed from 400 to 4,000 cm^–1^ at a resolution of 4 cm^−1^. The sample (2 mg) was mixed with KBr (400 mg) and then determined after grinding and tableting.

### Heteronuclear Single Quantum Coherence Spectroscopy

The isolated lignin was dissolved in 500 μL DMSO-d_6_ (∼50 mg) and then transferred into 5-mm NMR tube. The spectrum was acquired in a Bruker AV600 600 MHz NMR spectrometer with the ultra-low temperature QCI probe at 25°C. The heteronuclear single quantum coherence spectroscopy (HSQC) data were recorded by a Bruker adiabatic pulse program called “hsqcetgpsi2” with a spectral width of 9,615.4 Hz in F2(^1^H) and a spectral width of 3,3201.9 Hz in F1(^13^C). The original data were processed with MestReNova v14.0.

## Results and Discussion

### Growth and Lignin-Degrading Enzyme Activities of Fusants in Different Culture Medium

**FIGURE 1:**
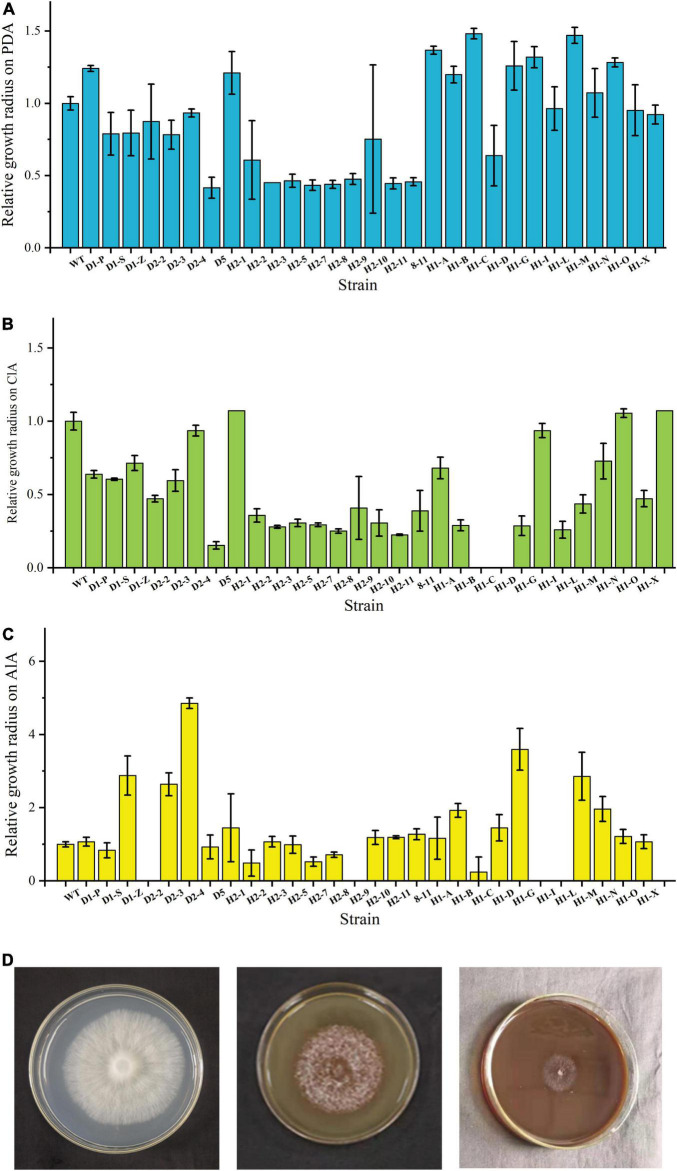
Growth status of WT and fusants on different plates. **(A)** Fusant/wild-type growth radius ratio histogram on PDA plates after incubation. **(B)** Fusant/wild-type growth radius ratio histogram on CLSS. **(C)** Fusant/wild-type growth radius ratio histogram on ALS. **(D)** Growth status of WT on PDA (left), CLSS (middle) and ALS (right).

To investigate which fusants can stably grow and utilize lignin as a carbon source for growth, WT and fusants were first cultured on different medium plates. Before this work was done, all fusants have been transferred on PDA plates for at least six rounds (data not shown). The results were shown in Figure 1A. All strains can stably grow on PDA plates. Some fusants show higher growth rate; then, WT and others also show different colony morphology (data not shown), indicating the occurrence of fusion in these strains. Among these fusants, D1-P, H2-1, H1-A, H1-B, H1-C, H1-G, H1-I, H1-M, and H1-O show a higher growth rate than WT on PDA plates.

These strains were also incubated on CLSS and ALS plates to analyze their capacity to degrade different lignin compounds. Some fusants did not grow on CLSS or ALS plates at all. H1-C and H1-D did not grow on CLSS; meanwhile, D2-2, H2-9, H1-I, and H1-L did not grow on ALS. Most fusants show a lower growth rate than WT. These results were in accordance with expectation as wide cross often leads to chromosome loss (Hara et al., 2012), which might influence the lignin degradation ability. H2-1 and H1-Y show a higher growth rate than WT on CLSS. D2-4 and H1-G show a higher growth rate than WT on ALS. These results were unexpected and show that fusion might lead to higher lignin utilization ability of these fusants (Figures 1B,C) and the growth status of WT on PDA, CLSS and ALS is shown in Figure 1D.

The growth status of strains in corn stalk was shown in Supplementary Figure 3. By comparing the weight loss of corn stalk caused by these strains, we can see that only WT and eight fusants (D1-P, D2-4, H2-1, H1-B, H1-C, H1-G, H1-I, and H1-Y) can vigorously grow in corn stalk and growing of these fusants led to higher weight loss of corn stalk than WT strain. Two fusants (8-11 and H1-X) degraded cellophane membrane and penetrate through it (data not shown). It was not possible to quantify the mycelium of these fusants added into the corn stalk; therefore, 8-11 and H1-X were not included in the following investigations.

To further investigate whether the growth of these fusants on lignin compounds or corn stalk was related to the presence of known lignin-degrading enzymes, the enzyme activity of lignin-degrading enzymes of all the fusants was tested after fermentation in liquid medium using calcium lignosulfonate or alkaline lignin as a sole carbon resource. Lac and MnP activity in ALL and CLSL of fusants and WT was determined by using ABTS and 2,6-DMP as substrate, respectively. It has been reported that Lac, MnP, versatile peroxidase (VP), and dye decolorizing peroxidase (DyP) have a similar mechanism in lignin degradation by generating free radicals (Xiao et al., 2020). The same substrate could be used to detect activity of MnP, VP, and DyP (Gao et al., 2017; Shrestha et al., 2017; Gaur et al., 2018; Hilgers et al., 2018). Lac is the main lignin degradation enzymes in *P. ostreatus* (Knežević et al., 2013). As the fermentation broth used in this experiment was not purified, the enzyme activity of Lac and MnP could represent a total activity of lignin degraded enzymes. The results were shown in Supplementary Figure 4. We can see that most fusants did not show enzyme activity, except for H1-G, H1-I, and H1-X in ALL and CLSL.

These findings suggest that protoplast fusion might lead to total or partial loss of the ability to degrade lignin and create fusants that can degrade lignin or lignocellulose without known lignin-degrading enzymes activity. Fusant D1-P was one of the candidate strains that can grow in medium with calcium lignosulfonate, alkaline lignin, and corn stalk without the help of known lignin-degrading enzymes activity. We also used three common laccase substrates that have been widely used to isolate laccase-producing microbes including RBBR, guaiacol, and tannic acid which were supplemented in PDA plate to further examine laccase activity in D1-P. The result shows that WT can oxidize all these substrates, but fusant D1-P cannot oxidize these substrates in PDA plates as represented in Supplementary Figure 5; therefore, it was selected for further investigation.

### Secreted Protein Mass Spectrometry During the Degradation of Corn Stalk Validates the Candidate Investigation Object D1-P

To validate the selection of D1-P for the following investigation, we first detected the enzyme activity with previous analysis. However, the dark brown color of the fermented liquor collected from corn stalk fermentation interfered with the detection process (data not shown). As all known lignin-degrading enzymes belong to secreted protein, we analyzed the total secreted protein of fusant D1-P and WT with LC-MS/MS. Samples were collected at three different fermentation time (7, 14, and 21 day) to include as many secreted proteins as possible. The results were represented in Tables 1, 2. A total of 887 proteins and 131 proteins were identified from WT and D1-P, respectively. Lac, MnP, VP, and DyP were all detected in the secreted protein of WT. However, no known lignin-degrading enzymes were detected in secreted protein of fusant D1-P. These findings suggest that D1-P does not able to produce known lignin-degrading enzymes at protein level. However, it could utilize calcium lignosulfonate, alkaline lignin, and corn stalk as carbon resources, meaning novel enzymes or mechanism might exist for the degradation of lignin.

**TABLE 1:** Secreted lignin-degrading enzyme proteins of wild type*.

Protein	Mass	Matches	Sequences	Coverage
LACC2	57,725	51 (44)	17 (16)	32%
VP2	38,908	40 (37)	7 (7)	35%
VP3	37,949	30 (27)	9 (9)	45%
LACC6	57,689	29 (27)	13 (12)	24%
DyP4	33,028	14 (13)	5 (5)	27%
MnP2	38,322	20 (20)	6 (6)	17%
MnP3	37,790	17 (12)	6 (5)	30%
MnP6	38,523	20 (16)	9 (9)	26%
MnP5	38,111	3 (3)	2 (2)	5%

*See the whole table in Supplementary Table 1.

**TABLE 2:** Partial secreted proteins of fusant D1-P*.

Protein	Mass	Matches	Sequences	Coverage
Glucanase_1038048	49,855	9 (9)	2 (2)	6%
Glucanase_1084743	56,791	10 (7)	3 (2)	7%
Uncharacterized protein	43,399	5 (5)	2 (2)	5%
Rhamnogalacturonan endolyase	54,684	4 (4)	1 (1)	3%
Serine proteinase	38,705	6 (4)	4 (3)	20%
Beta-galactosidase_1105441	110,709	4 (4)	2 (2)	1%
β- Xylanase	33,914	3 (3)	1 (1)	4%
Glycoside hydrolase family 31 protein	106,815	4 (4)	1 (1)	1%
Carboxylate hydrolase	59,747	2 (2)	1 (1)	2%
β- Galactosidase_1084879	33,022	3 (3)	1 (1)	4%

*See the whole table in Supplementary Table 2.

### D1-P Does Not Contain any Known and Functional Lignin-Degrading Genes

Even though lignin-degrading enzyme activity and protein peptides were not detected from D1-P strain, there is still the possibility that it is due to the improper induction conditions or the wrong sampling time. To exclude these possibilities, PCR was first used to verify the absence of known lignin-degrading enzyme genes in D1-P. The primers were designed based on the sequence information in known haploid strain *P. ostreatus* PC9 and PC15. Again, it was shown that no amplicon for all genes of lignin-degrading enzymes was obtained, further enhancing the credibility that these genes are absent in D1-P (Supplementary Figure 6).

As the wild-type strain used in this study was isolated from stalk, there is still the possibility that this strain might contain additional known lignin-degrading enzyme genes whose nucleic acid sequences were not identical to what has been found in model strain *P. ostreatus* PC9 and PC15. This might lead to omitting of these genes in PCR verification. To exclude this possibility, second-generation genome sequencing of fusant D1-P and a third-generation genome sequencing of WT were performed. By using genome sequencing technology, we analyzed the coding sequences (CDS) in WT and fusant D1-P (the full annotation of genes in fusant D1-P and WT was shown in Supplementary Tables 4, 5). As shown in Supplementary Table 5, all the genes about lignin degradation in WT were retrieved, and no additional lignin-degrading enzyme genes were discovered in strain 595 (WT).

Fusant D1-P is a recombinant strain created from WT and *Saccharomyces cerevisiae* which means all the lignin-degrading enzyme genes in fusant D1-P are from WT. We screened out the lignin-degrading enzyme genes in WT and then compared it with fusant D1-P to ensure every single known gene about lignin degradation in WT was destructed during the process of protoplast fusion. The results of comparison between WT and fusant D1-P are shown in Supplementary Table 6. Several laccase-like genes were obtained through BLAST search. The highest identity of all these laccase-like genes in D1-P is 80% to that of WT (Supplementary Table 6), but the align length (80/1626) is too short to ensure the gene can still express correct. These lignin-degrading enzyme genes in fusant D1-P underwent various degrees of recombination during protoplast fusion compared with WT.

### D1-P Retained Similar but Not Identical Lignocellulose Degradation Ability to WT

**FIGURE 2:**
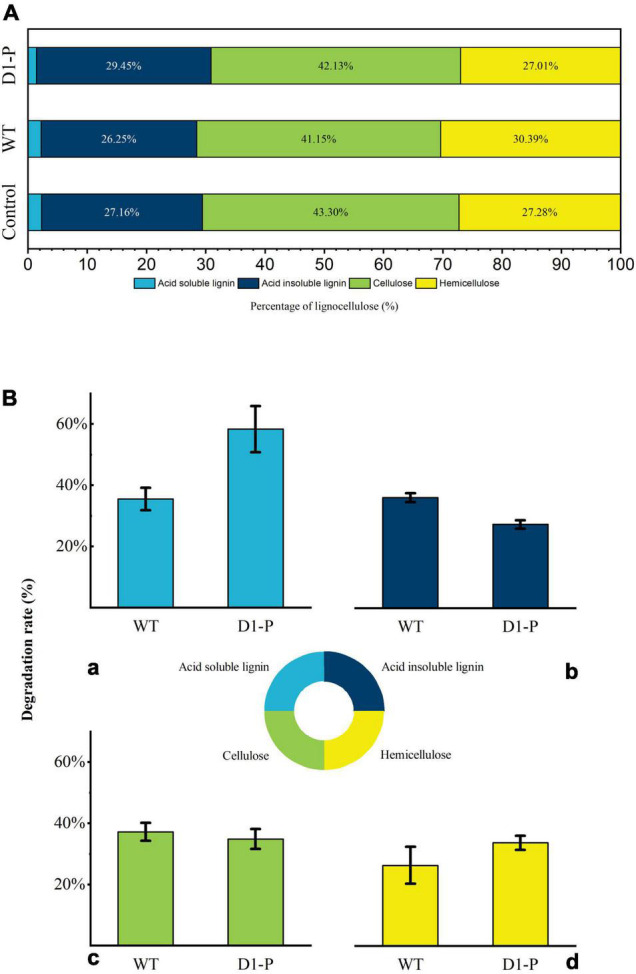
**(A)** Percentage of lignocellulose components in samples. **(B)** The degradation rate of lignocellulose by WT and D1-P determined by HPLC: (a) acid-soluble lignin; (b) acid-insoluble lignin; (c) cellulose; (d) hemicellulose.

The total weight loss of corn stalk caused by D1-P degradation might be attributed to its ability to consume hemicellulose or cellulose, not necessarily related to its lignin-degrading ability. To quantify the degrading ability of D1-P to different components in corn stalk, the degraded corn stalk was analyzed by the National Renewable Energy Laboratory (NREL) standard method (Sluiter et al., 2008). The degradation percentage of different lignocellulose components (cellulose, hemicellulose, acid-soluble lignin, and acid-insoluble lignin) in corn stalk was shown in Figure 2. After 4-week incubation, D1-P shows a similar degrading ability of cellulose and hemicellulose to that of WT. Without the known lignin-degrading enzymes, D1-P shows a lower degrading ability as compared to acid-insoluble lignin, which is the main component of lignin in corn stalk than WT (Figure 2A). This result confirms that traditional MnP DyP and Lac play certain roles in degradation lignin, whereas D1-P still retained about 85% of the lignin degradation ability without the help of these enzymes which probably explained why *in vitro* simulation experiment using these enzymes cannot successfully and efficiently degrade intact lignocellulose (Wariishi et al., 1991). Above all, it was interesting to find that the degrading ability of D1-P to acid-soluble lignin was significantly higher than WT. Although the content of acid-soluble lignin in softwood was very low, it was about 2%–10% in corn stalk (Guo et al., 2021). The structure of ASL also resembles to that of acid-insoluble lignin, meaning the increased degradation ability of D1-P to ASL probably lead to increased degradation ability to ASIL. These findings suggest that D1-P retained a similar total lignin-degrading ability to WT for corn stalk.

### D1-P Can Still Degrade Lignin Through Cleavage of β-O-4 Linkage

**FIGURE 3:**
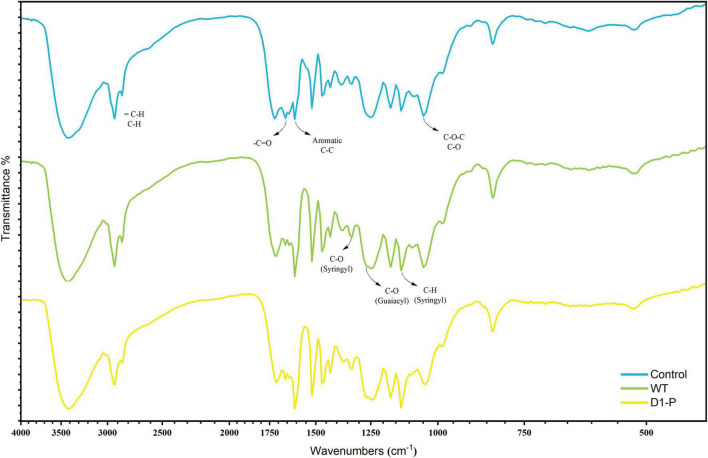
FTIR spectra of the milled lignin before and after degraded by WT and D1-P.

To uncover possible lignin-degrading mechanism in D1-P, the samples were first analyzed by FTIR. The FTIR spectra of MCSL, WMCSL, and DMCSL were shown in Figure 3, and the assignment of main absorption peaks was depicted in Supplementary Table 7 (Mansouri et al., 2011; Karmanov and Derkacheva, 2013; Joffres et al., 2014; Watkins et al., 2015). The strong absorption in all three samples around 3,400 cm**^–^**^1^ was caused by hydroxyl in carboxylic, phenolic, and alcoholic subunits of lignin and the remaining polysaccharide (cellulose and hemicellulose). An increased absorption was also observed in 1,262 cm**^–^**^1^ and 1,330 cm**^–^**^1^, which represent the stretching vibration of C-O in G (guaiacyl) unit and stretching vibration of C-O in S (syringyl) unit, respectively. A sharp absorption was observed in 1,605 cm**^–^**^1^ which indicated the increased free aromatic rings after the degradation due to the cleavage of lignin inter-linkage. The absorption around 1,655 cm**^–^**^1^ and 1,710 cm**^–^**^1^ was assigned to conjugated C = O and non-conjugated C = O. The non-conjugated C = O in 1,710 cm**^–^**^1^ was enhanced both in WMCSL and DMCSL because of the side-chain oxidation and β-O-4 linkage cleavage (Chen et al., 2016). Compared with the lignin after the degradation by WT, D1-P shows a lower absorption in 1,040 cm**^–^**^1^, representing the stretching vibration of C–O which was caused by β-O-4 linkage cleavage and WT did not show a significant selective degradation toward different kinds of linkage in lignin (which was further proved by HSQC analysis).

### D1-P Shows Cleavage Preferences to Substructure of β-O-4 Linkage Without the Help of Traditional Enzymes

**FIGURE 4:**
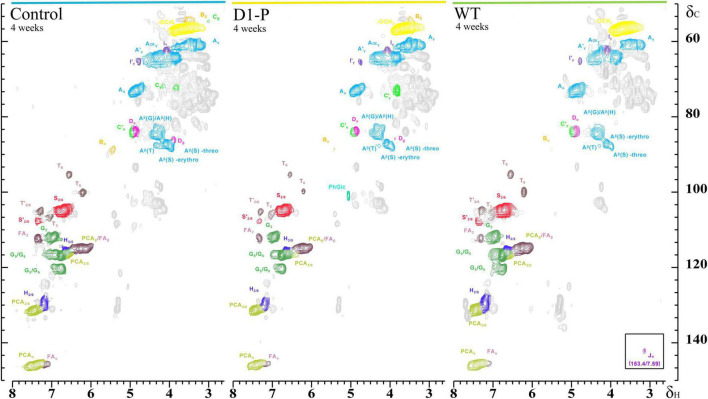
Side chain (δC/δH 50-90/2.5-5.8) and aromatic/unsaturated (δC/δH 90-155/5.5-8.0) regions in the 2D HSQC NMR spectra of isolated CEL (left), isolated DCEL (middle), and of the isolated WCEL (right).

**FIGURE 5:**
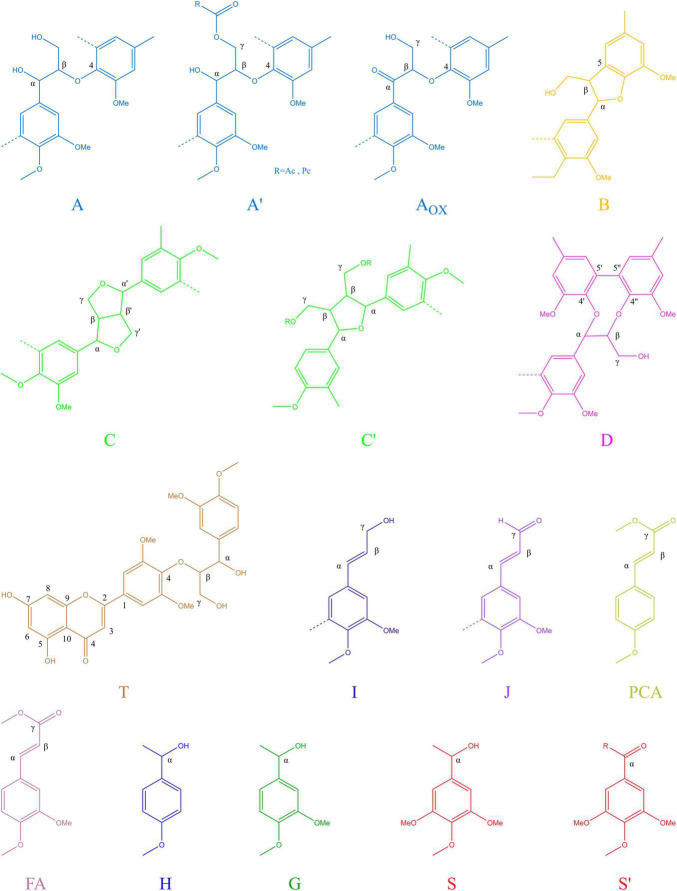
Main structures present in the lignins of corn stalk: (A) β-O-4′ alkyl-aryl ethers; (A′) β-O-4′ alkyl-aryl ethers with acylated γ-OH; (A_*ox*_) C_α_- oxidized β-O-4′ structures; (B) phenylcoumaran;(C) resinol; (C′) tetrahydrofuran; (D) dibenzodioxocins; (I) cinnamyl alcohol end groups; (J) cinnamyl aldehyde end groups; (PCA) p-coumarates; (FA) ferulates; (H) p-hydroxyphenyl units; (G) guaiacyl units; (S) syringyl units; (S′) oxidized syringyl units bearing a carbonyl at C_α_.

**FIGURE 6:**
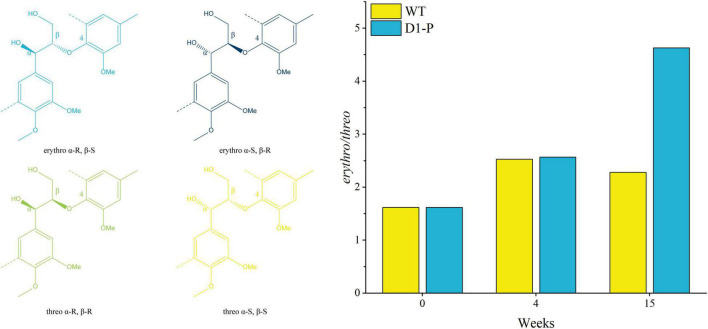
Ratio of β-O-4 *erythro/threo* after degrading by WT (yellow) and fusant D1-P (blue) determined by semi-quantitative HSQC NMR spectroscopy.

To further determine the structure changes in degraded lignin, we used HSQC technology to detect the chemical bonds in samples. The spectra of CEL, WCEL, and DCEL were colored and evaluated in Figure 4. The spectral of lignin appeared in δ_*C*_/δ_*H*_ 50-150/2.6-8.0. The signal in δ_*C*_/δ_*H*_ 50-120/2.6-5.5 shows the information of C-H correlation from aliphatic and that in δ_*C*_/δ_*H*_ 100-150/5.5-8.0 shows the information of C-H correlation from aromatic. The main structures in lignin were shown in Figure 5, and the details of assignment were shown in Table 3 (Ralph et al., 1994, 1999, 2004; del Río et al., 2008, 2011, 2012; Kim et al., 2008; Martínez et al., 2008; Rencoret et al., 2008, 2009; Ralph and Landucci, 2010; Ma et al., 2021). The strong signals in aliphatic area were generated by β-O-4 linkage in substructures of (A) including A_β_(G), A_β_(H), A_β_(S), and A_β_(T). The β-O-4 linkage in A_β_(G) and A_β_(S) formed a stereoisomerism presenting as two diastereomeric, threo, and erythro. Erythro and threo in A_β_(S) can be detected by HSQC, the A_β_(S)-*erythro* appeared at δ_*C*_/δ_*H*_ 86.56/4.08, and A_β_(S)-*threo* appeared at δ_*C*_/δ_*H*_ 87.12/3.97. Other structures were also clearly observed in spectrum including phenylcoumarans (B), resinols (C), tetrahydrofuran (C′), and dibenzodioxocins (D). After 4-week degradation by WT and D1-P, respectively, the lignin in stalk shows a different chemical bonds composition. To evaluate the changes in stalk, we performed a semi-quantitative analysis of the spectrum as shown in Table 4 (Martínez et al., 2008; Wen et al., 2010, 2013). Compared with control, WT shows a general degrading ability to nearly all kinds of lignin linkage and fusant D1-P also shows a degrading ability to β-O-4 linkage. Different to WT, fusant D1-P shows a selective degrading ability to the β-O-4 linkage in A_β_(S) for the different diastereomeric. After 4-week incubation, fusant D1-P degraded less A_β_(S)-*erythro* than A_β_(S)-*threo* and enriched this linkage. The other type of β-O-4 linkage in A_β_(G) and A_β_(H) was also degraded during the fermentation, but the diastereomers linked to G-units cannot be discriminated in HSQC spectrum. This mechanism has also been found in *Ceriporiopsis subvermispora*, another white-rot fungus. However, it has not been figured out which kind of lignin-degrading enzyme conducts this function (van Erven et al., 2019). To magnify the differences between WT and D1-P, we expanded the degradation time from 4 weeks to 15 weeks; then, HSQC was used to detect the stalk after degradation. The results of semi-quantitative analysis were shown in Supplementary Table 8. After 15-week degradation, the ratio of *erythro*/*threo* becomes bigger than 4-week degradation as shown in Figure 6. The signal of A_β_(G) and A_β_(H) was also observed a shape change in D1-P compared to control and WT in Supplementary Figure 7, and this may be caused by the degradation of diastereomers linked to G-units. *P. ostreatus* does not have LiP activity. After protoplast fusion with *S. cerevisiae*, fusant D1-P has lost all the known lignin-degrading enzyme genes which were proved by enzyme activity test and genome sequencing. This means that the selective lignin-degrading activity was not conducted by the traditional lignin-degrading enzymes. Notably after 4-week degradation, the percentage of H units was increased, and the S/G ratio was decreased in the degraded stalk. However, when degrading time was expanded to 15 weeks, the percentage of H units started to be lower and the S/G ratio becomes higher than control.

**TABLE 3:** Assignment of the lignin 13C-1H correlation peaks in 2D HSQC spectra of the corn stalk.

Label	δC/δH	Assignment
B_β_	53.6/3.47	C_β_-H_β_ in phenylcoumaran substructures (B)
C_β_	53.87/3.01	C_β_-H_β_ in β-β′ resinol substructures (C)
0	56.01/3.71	C-H in methoxyls
A_γ_	60.1/3.36 and 3.72	C_γ_-H_γ_ in γ-hydroxylated β-O-4′ substructures (A)
I_γ_	62.25/4.08	C_γ_-H_γ_ in cinnamyl alcohol end-groups (I)
A′_γ_	63.54/4.33	C_γ_-H_γ_ in γ-acylated β-O-4′ substructures (A′)
A′_γ_/Aox_γ_	63.54/3.86	Cγ^(^′^)^-Hγ^(^’^)^ in β-β′ resinol substructures (A′/Aox)
Aox_γ_	63.75/4.21	C_γ_-H_γ_ in γ-acylated β-O-4′ substructures (Aox)
I′_γ_	64.61/4.77	Cγ–Hγ in cinnamyl alcohol acylated at the γ-OH (I′)
C_γ_	71.22/4.14	Cγ-Hγ in β-β′ resinol substructures (C)
C_γ_/C′_γ_	71.5/3.82	Cγ^(^’^)^-Hγ^(^′^)^ in β-β′ resinol substructures (C/C′)
A_α_	72.14/4.86	C_α_-H_α_ in β-O-4′ substructures (A) linked to a G-unit
D_α_	83.11/4.88	C_α_-H_α_ in dibenzodioxocin substructures (D)
C′_α_	83.43/4.95	C_α_–H_α_ in β-β (C′, tetrahydrofuran)
A_β_(G)/ A_β_(H)	83.97/4.35	C_β_-H_β_ in β-O-4′ substructures (A) linked to a G/H unit
D_β_	85.52/3.89	C_β_-H_β_ in dibenzodioxocin substructures (D)
A_β_(S) -erythro	86.56/4.08	C_β_–H_β_ in β–O–4 linked to S (A, Erythro)
A_β_(T)	86.81/4.34	C_β_–H_β_ in β–O–4 linked to T
A_β_(S) -threo	87.12/3.97	C_β_–H_β_ in β–O–4 linked to S (A, Thero)
Bα	87.87/5.43	C_α_-H_α_ in phenylcoumaran substructures (B)
T_8_	94.73/6.56	C_8_–H_8_ in tricin (T)
T_6_	99.46/6.21	C_6_–H_6_ in tricin (T)
PhGlc	100.11/5.05	phenyl glycoside
S_2/6_	103.98/6.7	C_2_-H_2_ and C_6_-H_6_ in etherified syringyl units (S)
T′_2,6_	104.63/7.31	C′_2,6_–H′_2,6_ in tricin (T)
T_3_	105.7/7.04	C_3_–H_3_ in tricin (T)
S′_2/6_	106.99/7.39	C_2_-H_2_ and C_6_-H_6_ in etherified syringyl units (S′)
G_2_	111.29/6.95	C_2_-H_2_ in guaiacyl units (G)
FA_2_	111.51/7.34	C_2_-H_2_ in ferulate (FA)
PCA_β_ and FA_β_	114.09/6.24	C_β_-H_β_ in p-coumarate (PCA) and ferulate (FA)
H_3,5_	114.81/6.67	C_3_-H_3_ and C_5_-H_5_ in p-coumarate (H)
PCA_3,5_	115.73/6.6	C_3_-H_3_ and C_5_-H_5_ in p-coumarate (PCA)
G_5_/G_6_	116.03/6.76 and 118.82/6.76	C_5_-H_5_ and C_6_-H_6_ in guaiacyl units (G)
H_2,6_	128.72/7.2	C_2,6_-H_2,6_ in p-hydroxyphenyl units (H)
PCA_2,6_	130.65/7.43	C_2_-H_2_ and C_6_-H_6_ in p-coumarate (PCA)
FA_α_	145.05/7.12	C_α_-H_α_ in ferulate (FA)
PCA_α_	145.28/7.4	C_α_-H_α_ in p-coumarate (PCA)
J_α_	153.9/7.57	C_α_-H_α_ in cinnamyl aldehyde end-groups (J)

**TABLE 4:** Structural characteristics (lignin interunit linkages, aromatic units, and S/G ratio, p-Coumarate/Ferulate, tricin) from integration of C-H correlation peaks in the HSQC spectra of the corn stalk after 1-month degradation.

	Control	WT	D1-P
**Lignin Interunit linkages (%)**			
β-O-4′ substructures (A/A′)	52.72%	31.09%	49.46%
β-O-4′ substructures - A_β_(G)/A_β_(H)	31.43%	21.21%	28.97%
β-O-4′ substructures - A_β_(S) - *erythro*	13.13%	6.70%	14.16%
β-O-4′ substructures - A_β_(S) - *threo*	7.82%	2.64%	5.51%
β-O-4′ substructures - A_β_(T)	0.33%	0.53%	0.83%
β-5′ phenylcoumaran substructures (B)	1.07%	ND	0.85%
β-β′ resinol substructures (C)	1.54%	ND	ND
Tetrahydrofuran (C′)	1.75%	2.12%	1.19%
Dibenzodioxocins (D)	12.33%	6.20%	11.40%
**Lignin Aromatic Units*^a^***			
H (%)	9.53%	23.16%	12.21%
G (%)	39.14%	34.21%	39.45%
S (%)	51.33%	42.63%	48.34%
S/G ratio	1.31	1.25	1.23
***p*-Hydroxycinnamates*^b^***			
*p*-coumarates (%)	49.24%	36.60%	45.79%
ferulates (%)	10.45%	3.02%	6.12%
*p*-coumarates/ferulates ratio	4.7	12.1	7.5
**Flavonoid*^b^***			
tricin (T)	4.36%	4.71%	1.99%

*^a^Molar percentages (H + G + S = 100).*

*^b^Interunit linkages, with p-coumarate, ferulate, cinnamyl alcohol end-groups, cinnamyl aldehyde end-groups and tricin molar contents as percentages of lignin content (H + G + S).*

## Conclusion

By wide cross between *P. ostreatus* and *S. cerevisiae*, a serial fusants were produced to further discover novel lignin degradation mechanism. After serious screening, fusant D1-P was proved not able to synthesize traditional lignin-degrading enzymes in protein level and gene level. However, D1-P can still degrade lignin without the known lignin-degrading enzymes in *P. ostreatus*. Further investigation shows that D1-P can still degrade lignin in corn stalk by oxidative cleavage of β-O-4 linkage. It also shows the selective degradation to β-O-4 linkage in A_β_(S)-*threo* without the help of traditional lignin degradation enzymes. These results indicate that wide cross is powerful tool to uncover novel lignin degradation mechanism. These results will certainly contribute to the understanding of lignin degradation mechanism and improving the valorization of lignocellulosic biomass in future.

## Data Availability Statement

The final assembled genomes of *P. ostreatus* 595 has been deposited to NCBI with an accession code: JAKNCV000000000. The raw Nanopore reads of *P. ostreatus* 595 have been uploaded to Sequence Read Archive (SRA) and the submission code is SRX15507623. The final assembled genomes of fusant D1-P has been deposited to NCBI with an accession code: JAMQCK000000000. The raw Illumina reads of fusant D1-P have been uploaded to Sequence Read Archive (SRA) and the submission code is SRX15498264.

## Author Contributions

HF: conceptualization, resources, methodology, and funding acquisition. DW: methodology, formal analysis, data curation, and writing—review and editing. QS: investigation, methodology, formal analysis, and draft manuscript writing. XL, YCh, ZZ, and YCu: investigation. All authors contributed to the article and approved the submitted version.

## Conflict of Interest

YCu was employed by Tianjin Tianren Century Technology Co., Ltd., Tianjin, China. The remaining authors declare that the research was conducted in the absence of any commercial or financial relationships that could be construed as a potential conflict of interest.

## Publisher’s Note

All claims expressed in this article are solely those of the authors and do not necessarily represent those of their affiliated organizations, or those of the publisher, the editors and the reviewers. Any product that may be evaluated in this article, or claim that may be made by its manufacturer, is not guaranteed or endorsed by the publisher.

## Supplementary Material

Supplementary Figure 1 Analyze procedure of lignin-degrading enzyme genes in WT and D1-P.

Supplementary Figure 2 Isolation procedure of cellulolytic enzyme lignin (CEL) and milled corn stalk lignin (MCSL) from corn stalk degraded by WT and D1-P.

Supplementary Figure 3 Growth status in corn straw medium: **(A)** The control group is the medium without inoculation. **(B)** Wild-type strain; **(C–J)** D1-P, D2-4, H2-1, H1-B, H1-C, H1-G, H1-I, H1-Y strains. **(K)** The dry weight loss ratio of fusant/wild-type strain cultured with straw as a medium.

Supplementary Figure 4 Lignin-degrading enzyme activity in different culture medium: **(A)** Enzyme activity of MnP in fermentation broth with calcium lignosulfonate as the sole carbon source. **(B)** Enzyme activity of Lac in the fermentation broth with calcium lignosulfonate as the sole carbon source. **(C)** Enzyme activity of MnP in fermentation broth with alkali lignin as the sole carbon source. **(D)** Enzyme activity of Lac in the fermentation broth with alkali lignin as the sole carbon source.

Supplementary Figure 5 Laccase detection in D1-P and WT with well-recognized substrates. Guaiacol (left), RBBR (middle), and tannic acid (right).

Supplementary Figure 6 Amplification of lignin-degrading enzyme genes from WT and D1-P strains. **(A)** Marker (lane M) and genome of WT (lane 1) and fusant D1-P (lane 2). **(B)** Positive control of WT (lane 1) and fusant D1-P (lane 2). **(C)** Amplification of MnP Lac and VP genes in the genome of WT. **(D)** Amplification of MnP Lac and VP genes in the genome of fusant D1-P. Lanes M to 20 represent Marker, MNP1, MNP2, MNP3, MNP4, MNP5, LAC1, LAC2, LAC3, LAC4, LAC5, LAC6, LAC7, LAC8, LAC9, LAC10, LAC11, LAC12, VP2, and VP3, respectively, and the predetermined sizes of PCR products are 833 bp, 731 bp, 585 bp, 612 bp, 645 bp, 449 bp, 582/584 bp, 655 bp, 739/744 bp, 617/619 bp, 553 bp, 538 bp, 701 bp, 544 bp, 579 bp, 632/627 bp, 772 bp, 519 bp, 600 bp, and 703 bp, respectively.

Supplementary Figure 7 Side chain (δ_*C*_/δ_*H*_ 50-90/2.5-5.8) and aromatic/unsaturated (δ_*C*_/δ_*H*_ 90-155/5.5-8.0) regions in HSQC NMR spectra of MCSL (left), DMCSL (middle), and WMCSL (right).
