# Supplementary figures and images for: Investigations on the Fusants From Wide Cross Between White-Rot Fungi and Saccharomyces cerevisiae Reveal Unknown Lignin Degradation Mechanism
Source: Front Microbiol. 2022 Jul 11;13:935462. doi: 10.3389/fmicb.2022.935462 (PMC9310788; doi:10.3389/fmicb.2022.935462)

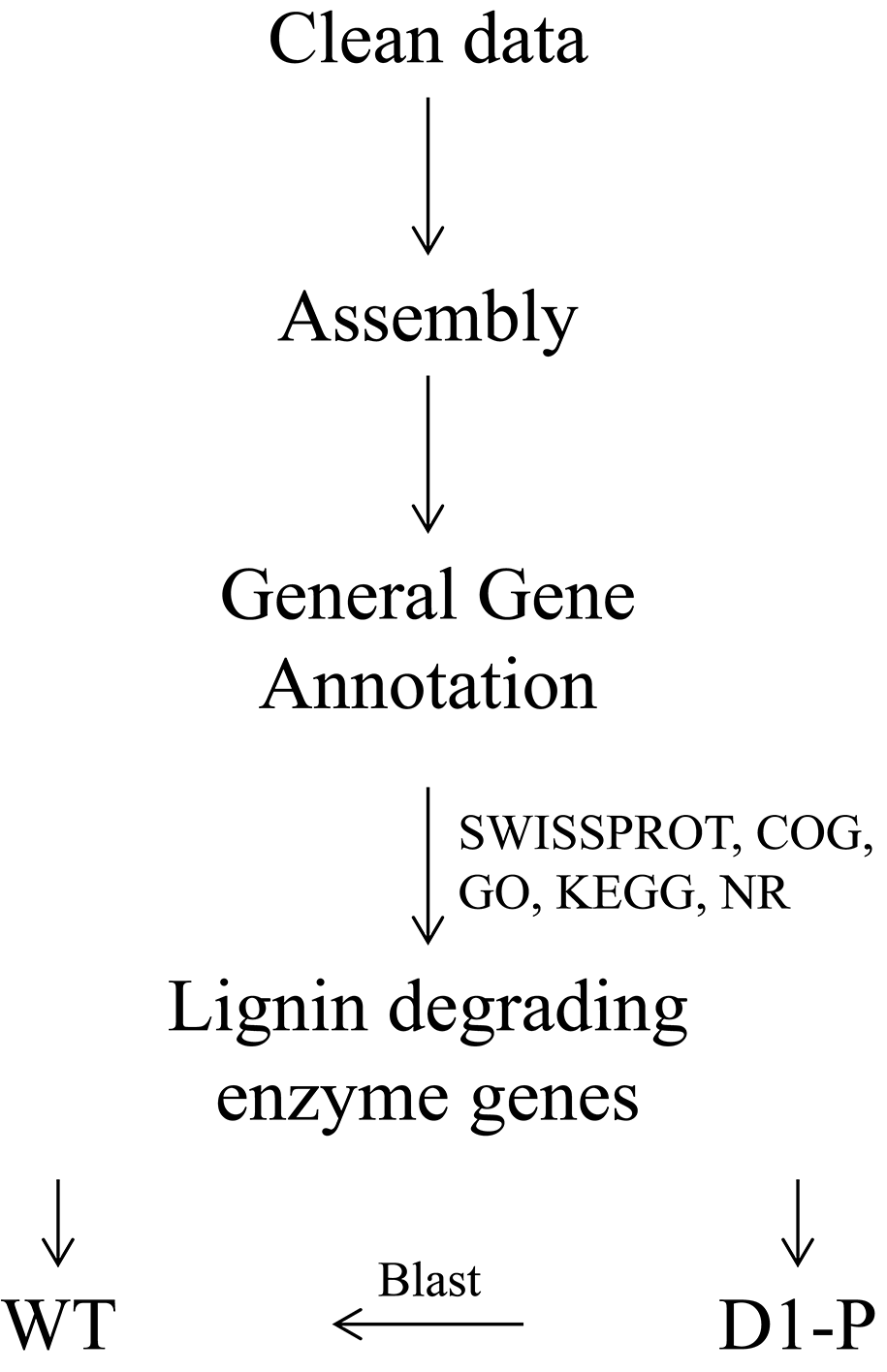

Supplement: Supplementary Figure 1 — Analyze procedure of lignin-degrading enzyme genes in WT and D1-P. [file Image_1.TIF]

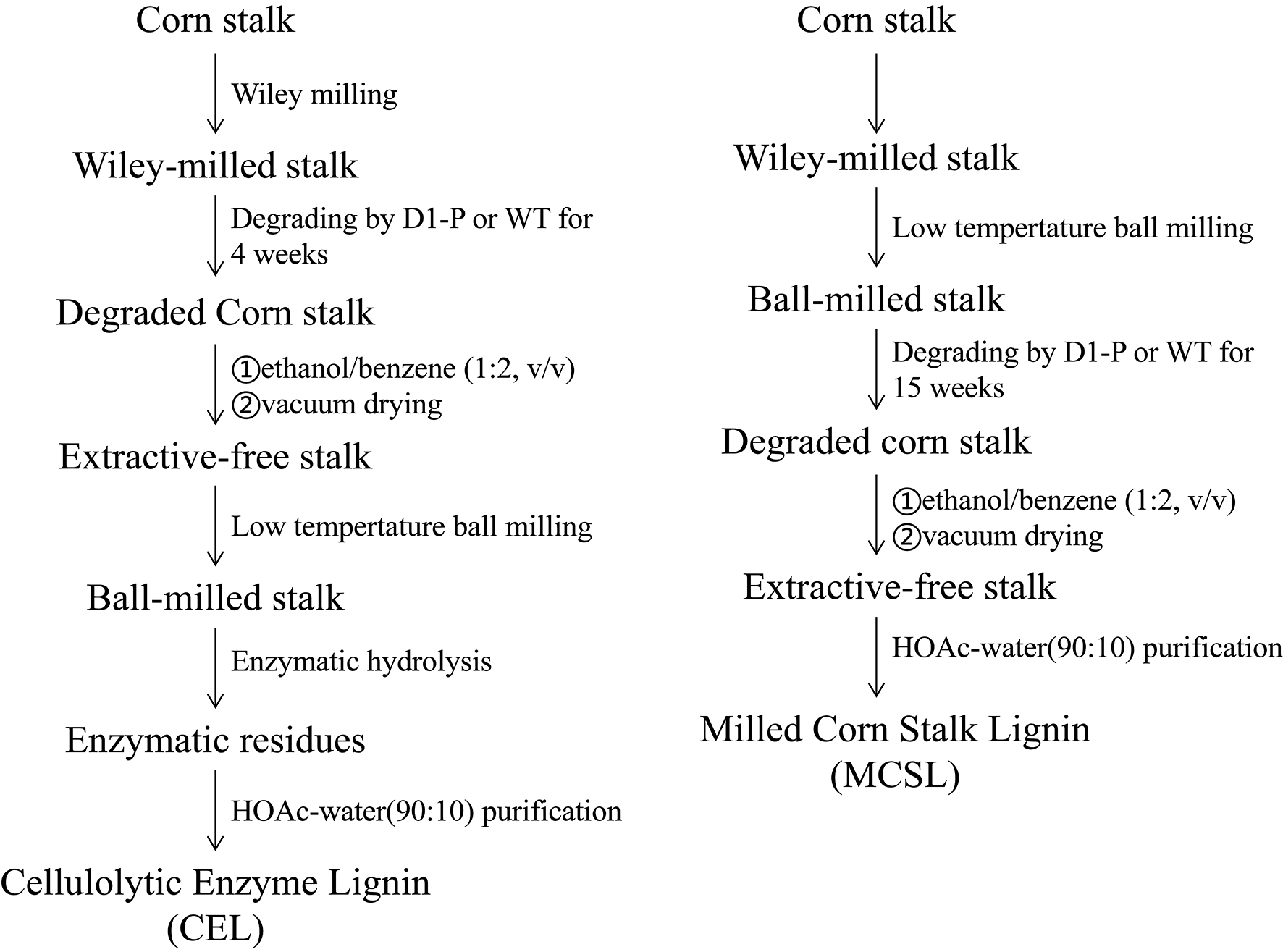

Supplement: Supplementary Figure 2 — Isolation procedure of cellulolytic enzyme lignin (CEL) and milled corn stalk lignin (MCSL) from corn stalk degraded by WT and D1-P. [file Image_2.TIF]

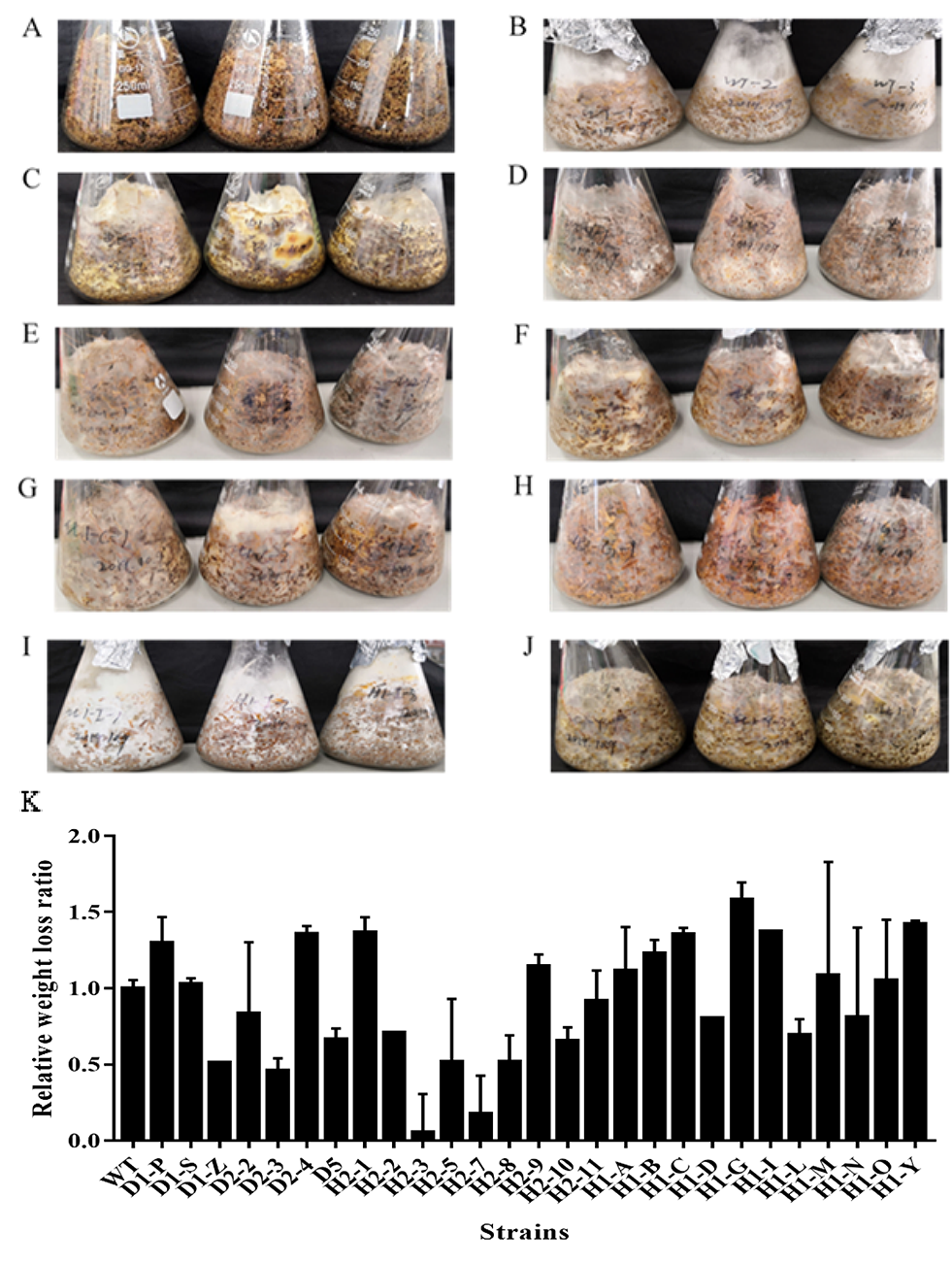

Supplement: Supplementary Figure 3 — Growth status in corn straw medium: (A) The control group is the medium without inoculation. (B) Wild-type strain; (C–J) D1-P, D2-4, H2-1, H1-B, H1-C, H1-G, H1-I, H1-Y strains. (K) The dry weight loss ratio of fusant/wild-type strain cultured with straw as a medium. [file Image_3.TIF]

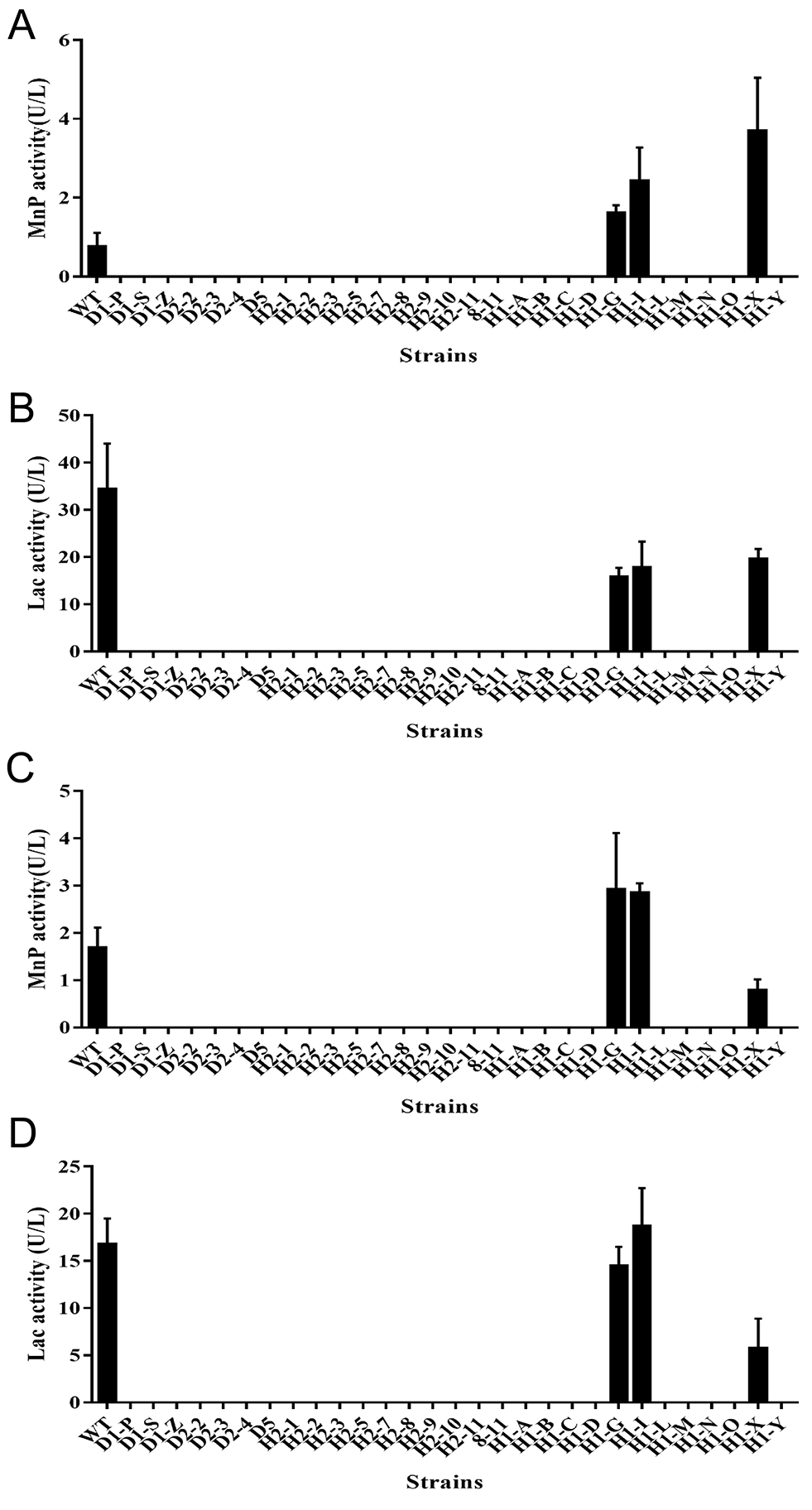

Supplement: Supplementary Figure 4 — Lignin-degrading enzyme activity in different culture medium: (A) Enzyme activity of MnP in fermentation broth with calcium lignosulfonate as the sole carbon source. (B) Enzyme activity of Lac in the fermentation broth with calcium lignosulfonate as the sole carbon source. (C) Enzyme activity of MnP in fermentation broth with alkali lignin as the sole carbon source. (D) Enzyme activity of Lac in the fermentation broth with alkali lignin as the sole carbon source. [file Image_4.TIF]

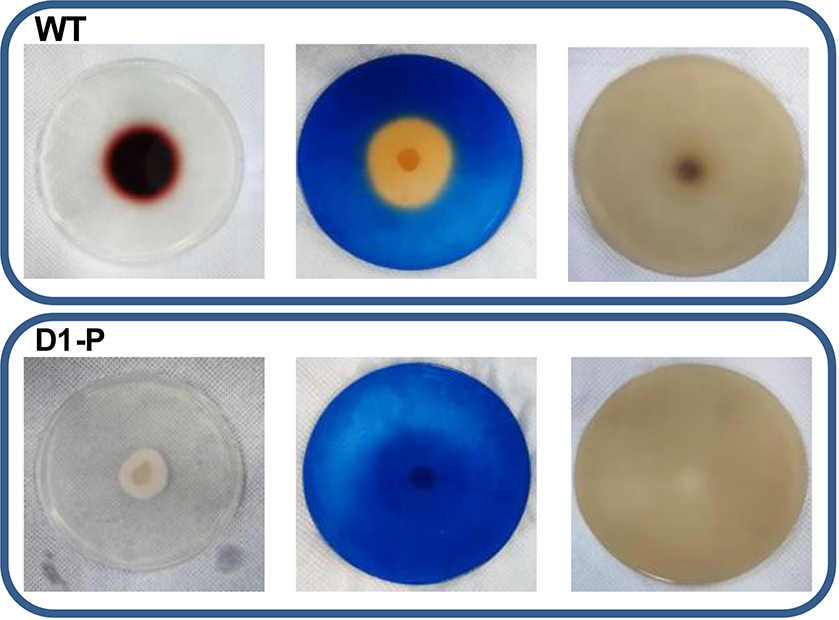

Supplement: Supplementary Figure 5 — Laccase detection in D1-P and WT with well-recognized substrates. Guaiacol (left), RBBR (middle), and tannic acid (right). [file Image_5.TIF]

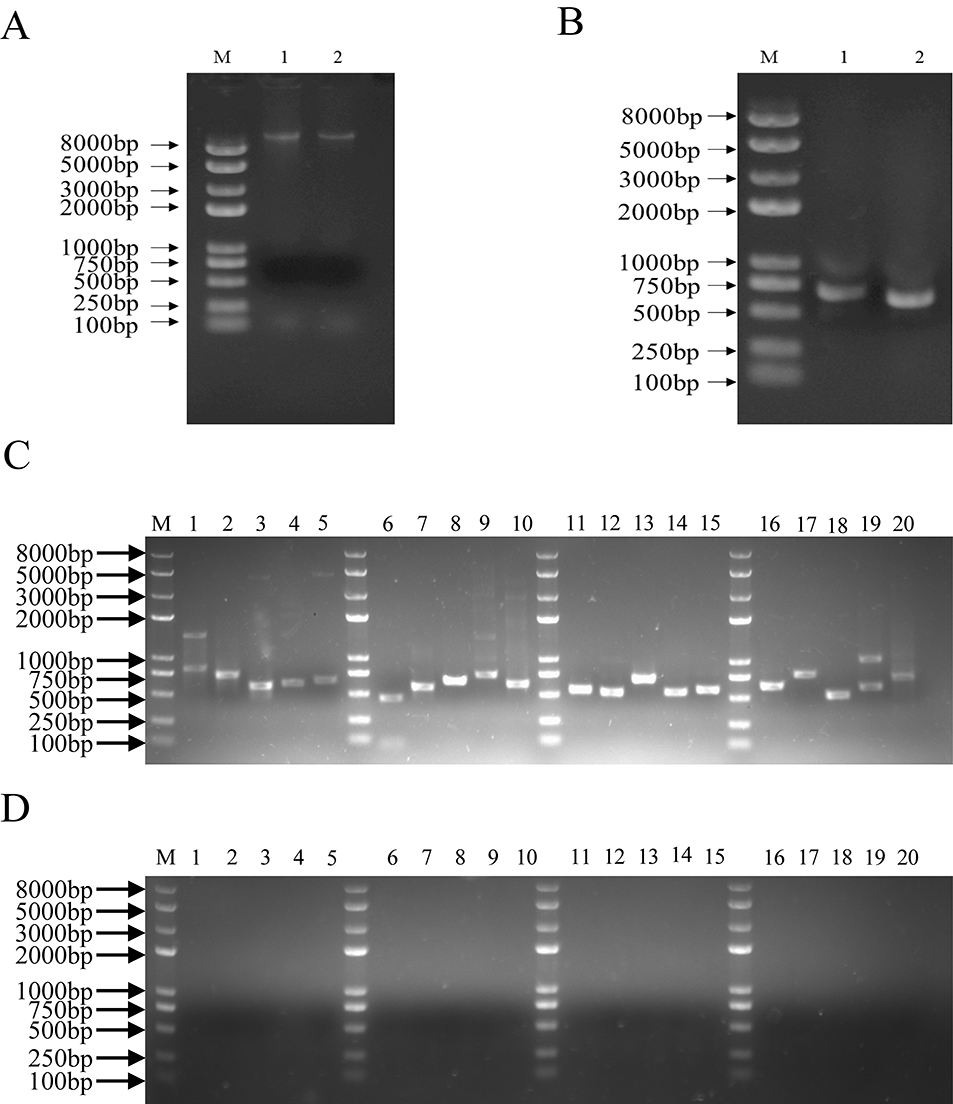

Supplement: Supplementary Figure 6 — Amplification of lignin-degrading enzyme genes from WT and D1-P strains. (A) Marker (lane M) and genome of WT (lane 1) and fusant D1-P (lane 2). (B) Positive control of WT (lane 1) and fusant D1-P (lane 2). (C) Amplification of MnP Lac and VP genes in the genome of WT. (D) Amplification of MnP Lac and VP genes in the genome of fusant D1-P. Lanes M to 20 represent Marker, MNP1, MNP2, MNP3, MNP4, MNP5, LAC1, LAC2, LAC3, LAC4, LAC5, LAC6, LAC7, LAC8, LAC9, LAC10, LAC11, LAC12, VP2, and VP3, respectively, and the predetermined sizes of PCR products are 833 bp, 731 bp, 585 bp, 612 bp, 645 bp, 449 bp, 582/584 bp, 655 bp, 739/744 bp, 617/619 bp, 553 bp, 538 bp, 701 bp, 544 bp, 579 bp, 632/627 bp, 772 bp, 519 bp, 600 bp, and 703 bp, respectively. [file Image_6.TIF]

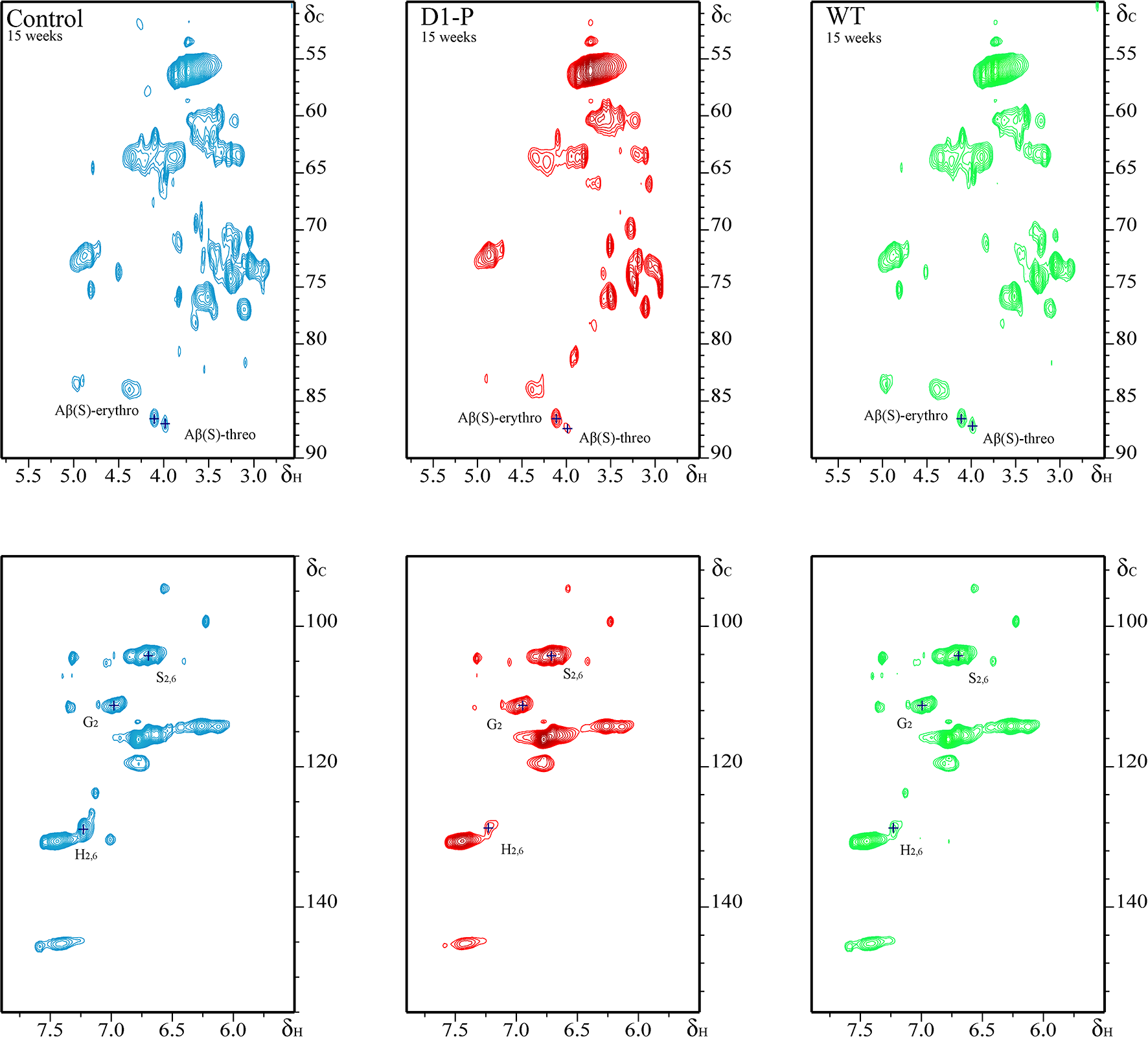

Supplement: Supplementary Figure 7 — Side chain (δC/δH 50-90/2.5-5.8) and aromatic/unsaturated (δC/δH 90-155/5.5-8.0) regions in HSQC NMR spectra of MCSL (left), DMCSL (middle), and WMCSL (right). [file Image_7.TIF]
